# An Imbalance Regression Approach to Toxicity Prediction of Chemicals for Potential Use in Environmentally Acceptable Lubricants

**DOI:** 10.1021/acsami.4c10622

**Published:** 2025-03-10

**Authors:** B. Al-Jubouri, I. Desiati, W. Wijanarko, N. Espallargas

**Affiliations:** a The Norwegian Tribology Center, Department of Mechanical and Industrial Engineering, Norwegian University of Science and Technology (NTNU), R. Birkelandsvei 2B, Trondheim 7491, Norway; b Department of Computer Science, York St John University, Lord Mayor’s Walk, York, York YO31 7EX, United Kingdom

**Keywords:** environmentally acceptable lubricants, toxicity, machine learning, imbalance regression, molecular descriptors

## Abstract

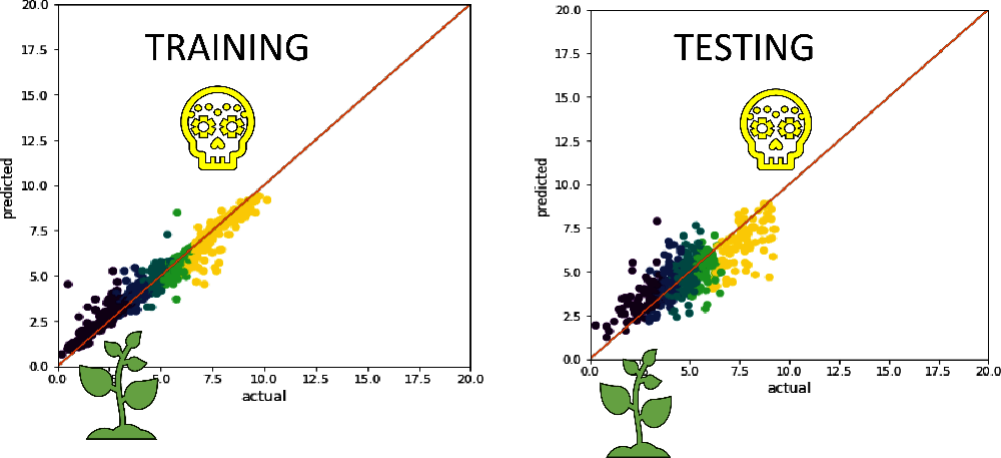

Lubricants are complex mixtures of chemicals that help machines function at the right level of friction and wear. Lubricant formulation methods are based on empirical experience of chemical substances that have been used as lubricants for decades. In the last years, the discussion about their environmental problem has triggered new legislations resulting in the search for Environmentally Acceptable Lubricants, which should be biodegradable, minimally toxic, and nonbioaccumulative. Finding new chemicals that comply with these three criteria is a long and expensive process that can be boosted by machine learning (ML). In this paper, we are addressing toxicity prediction with machine learning models by exploring the application of ensemble learners to chemicals having imbalanced data distribution. We investigated the effectiveness of sampling techniques to balance the data and improve the performance of the ensemble learning model. The model can predict toxicity for nonundersampled groups, which in our case corresponds to the moderately to highly toxic groups. The results of this work are useful for lubricant formulators since regulations accept moderate-to-highly toxic chemicals in lubricants if their concentration is below 20 wt %.

## 1 Introduction

Lubricants are used for achieving the required level of friction and wear in moving systems. They can be either liquid, solid, or semisolid (greases). Their formulation is rather simple, i.e., it consists of a base fluid in the highest proportion (up to 95%) and an additive package (chemical substances that provide the main functionality and improve its properties). Additives can radically change the properties of a lubricant and are essential to its overall performance. Lubricants can be classified depending on the nature of the base fluid, biological and nonbiological. This already accounts for a vast collection of compounds, mostly hydrocarbons. The base fluid can control wear, friction, and other characteristics such as toxicity, but the additive package is the main factor responsible for the overall function of the lubricant and for accounting for its chemical stability.

Lubricant formulation and production are a century-old industry in which the development of formulations is still based on existing recipes that add selected additives. Current state of the art for producing lubricants is based on empirical experience of substances (liquid or solid) that have been functioning as lubricants for decades. In addition, the additives giving functionality and improving lubricant performance are typically found by trial-and-error development. An example of this is the well-known zinc dialkyldithiophosphate (ZDDP), which was originally developed as antioxidant and finally turned out to be the best additive for wear control.^1,2^ This approach brings only small incremental improvements to lubricant formulations.

Nowadays environmental concerns are pushing the lubricant industry to move toward coping with the new societal demands on a greener economy. Europe is responsible for 19% of lubricants demand, consuming 6.8 million tons of lubricants every year.^3^ In the EU and in the world, about 50% of lubricants purchased end up as waste (the remaining 50% is burned or lost during the year).^4^ Therefore, the EU manages about 3.5 million tons per year of Waste Lubricating Oil (WLO). WLO is hazardous to public health and the environment because it contains high concentrations of toxic and carcinogenic substances, such as heavy metals, polychlorinated hydrocarbons, polyaromatic compounds, etc. The environmental effects of waste lubricant cause pollution, from leaks, losses, combustion, or dumping, to both on the soil, the water, and the air. Specifically, one liter of WLO can contaminate up to one million liters of drinking water, plants uptake contaminants in the soil, and burning WLO releases more than 50% of lead, chromium, and zinc in the form of particles.^4,5^ Therefore, the development of the greener generation of lubricants is happening at lower speed than required by the new environmental legislations.^6^

To cope with new legislation, a new generation of lubricants, the so-called Environmentally Acceptable Lubricants (EALs), should be developed faster. EALs should both be biodegradable, nontoxic, and nonbioaccumulative. Finding new chemicals that comply with these characteristics is a long and expensive process that can be boosted with the help of machine learning (ML). Especially in toxicity prediction, machine learning models can provide quick suggestions for chemical compounds with no potential harmful effects to the environment.

Machine learning has been applied in several fields of tribology, ranging from composite to steel materials, drive technology to manufacturing, and also lubricants.^7^ A few examples of machine learning applications in this field include predicting band gaps using artificial neural networks (ANN)^8^ and tuplewise graph neural networks.^9^ Additionally, ANN has been employed to predict topological phases of matter,^10^ while support vector machines, multiple linear regression, and ensemble trees have been used to predict exfoliation energies.^11^ One of the main challenges in the tribology field that is common to other engineering fields is collecting quality data. It can be difficult to obtain good size data sets with similar experimental settings.

Previous works using machine learning to predict the toxicity of chemical compounds have been mainly carried out in drug discovery.^12−16^ In these studies, toxicity prediction is often considered as a classification problem, where the aim is to predict whether a certain chemical compound is toxic or not. However, in available data sets for compounds considered in lubricant generation, toxicity is measured as a continuous value. Both highly toxic chemicals and nontoxic chemicals are less reported in such data sets, which results in imbalance target distribution. Regression models are used to predict continuous values; however, the problem of imbalance target distribution is less studied in regression than in classification. In regression problems, target values are treated with equal importance, and the model is evaluated and optimized based on the most common values in the target distribution.

This presents two challenges that this paper will address: first to develop a model that can accurately predict continuous toxicity values and second to account for the imbalance in the toxicity target value. Preprocessing techniques are carried out to prepare and balance the data, and an ensemble model, namely, eXtremeGradient Boosting (XGBoost), is used to provide the toxicity value prediction. Our main aim is to thoroughly investigate the challenges encountered in predicting toxicity targeting lubricants, and to this extend, this paper will:

1. Explore the application of ensemble learners to predict the toxicity value for different types of chemicals that have imbalanced data distribution.
2. Investigate the effectiveness of sampling techniques to balance the data and improve the performance of the ensemble learning model.
3. Compare two different types of chemical descriptor generators, namely, Morgan FingerPrints (MFP) and descriptors generated by a commercial software (AlvaDesc).

## 2 Methods

### 2.1 Data Collection

An experimental database for aquatic toxicity was retrieved from ECOTOX database as of March 2023.^17^ The database contained 1,141,099 experimental results from 12,732 chemicals using 13,864 species. The ECOTOX database was collected from 53,927 references. This is the initial database, which will be further curated and filtered to include only the toxicity tests of chemicals performed on water flea.

#### 2.1.1 Curation of the Molecular Structures

Chemical Abstracts Service Registry Numbers (CASRN) were used as queries to retrieve chemical information, such as chemical name, chemical formula, molecular mass, International Chemical Identifier code (InChiKey), and Simplified Molecular-Input Line-Entry System (SMILES). This chemical information was retrieved via CompTox Chemicals Dashboard.^18^ The queries generated 12,792 chemical records from 12,732 CASRN inputs, which were then manually checked. It was found that 60 CASRN generated a record twice. All records that had a mismatch CASRN were deleted. In addition, 1794 chemical records with no molecular weight information and 2 chemical records with no SMILES information were deleted. In total, 10,936 chemical information corresponding to 10,936 chemicals were retained. Retrieving back 10,936 chemicals from the ECOTOX database resulted in a cured database containing 1,076,925 experimental results using 13,584 species from 52,193 references.

#### 2.1.2 Data Filtering

The cured database includes data from several test conditions, such as test locations (laboratory or field), exposure media (water or soil), and exposure types (both aquatic and terrestrial or aquatic only). The cured database also includes data from acute and chronic toxicity tests with various species groups. Acute toxicity tests are performed as a short-term exposure to several concentrations of the chemical. Two end points were normally used for acute toxicity test, i.e., Lethal Concentration (LC50) and Effective Concentration (EC50) in mg/L. LC50 is defined as the concentration of the chemical in water causing 50% of death of the test species population, and EC50 is the concentration of chemical in water to produce a certain effect in 50% of the test population. Chronic toxicity tests are performed by long-term exposure of the species to the chemical. Several end points were used for the chronic toxicity test, such as No Observed Effect Concentration (NOEC) and Lowest Observed Effect Concentration (LOEC). The tested species groups in the database consist of animals and plants such as amphibians, crustaceans, fish insects, algae, fungi, etc. Workflows using Rstudio (v2023.03.0) programming language were used to extract LC50 or EC50 (L(E)C50) values for water flea tested in lab over a test duration of 48 h based on The Organization for Economic Co-operation and Development (OECD) test Guideline number 202. The resulting water flea database now contained 9705 L(E)C50 experimental values from 1863 chemicals, meaning several chemicals were tested in more than one experiment. In the case of chemicals with more than one experiment, the median L(E)C50 values were selected because they are not affected by low or high extreme values. Then, all median L(E)C50 values were transformed to a logarithmic scale (−Log mol/L). One outlier data was discharged due to a high log value. The resulting database filtered from the cured database contained 1862 chemicals with the corresponding CASRN and −Log(L(E)C50). From the 1862 chemicals, 1331 chemicals are organic compounds, 50 are inorganic compounds, 422 are ionic compounds, and 59 are mixture compounds.

Besides the database obtained from ECOTOX, a database from a previous study that was published in ref (16) was collected for comparison. This database was selected based on having a comparative study performed on toxicity of chemicals; however, that work does not specifically target lubricants. We have not found any work performed on predicting the toxicity of lubricants or any other environmental acceptability descriptor for lubricants using machine learning. This database is referred to as the “ITA database” and consists of 546 organic chemicals with toxicity Log(LC50) values as well as CASRN and SMILES information. Due to the availability of logarithmic values and CASRN, the ITA database did not go through our molecular structure curation protocol.

#### 2.1.3 Data Set Preparation

Three data sets were prepared for this study, namely, the ECOTOX chemicals (referred as “All” data set), the ECOTOX organic chemicals that do not overlap with the ITA data set (referred as “Clean” data set), and the ITA data set alone.

Before generating the molecular descriptors, all chemicals in the data sets were checked for their molecular objects. Molecular objects are algorithms that store all information related to the molecular structure geometry and topology. This was done in Python using the RDkit package (v2023.03.1). The chemicals that cannot generate molecular objects were removed from the data set. Nine chemicals were removed from the “All” data set, four chemicals were removed from the “Clean” data set, and one chemical was removed from the ITA data set. In the end, the “All” data set consisted of 1853 chemicals, the “Clean” data set consisted of 909 chemicals, and the ITA data set consisted of 545 chemicals.

### 2.2 Molecular Descriptor Generation

To predict the toxicity of molecules using machine learning (ML), the molecules should be represented in a numeric format for the computer to understand the input. Molecules in the machine-readable format can thus be passed to the learning algorithms while still capturing the comprehensive structure. Selecting a proper molecular representation that corresponds with the data set is essential for downstream analysis.

Molecules can be represented in several ways, mostly based on their feature representation. The simplest way to describe molecules is by listing their physicochemical characteristics in a numeric format or commonly addressing them by the molecular descriptor. These descriptors, such as molecular weight, density, polar surface area (PSA), hydrophobicity, etc., define the molecules’ structure–activity that interact with the biological environment as well as their molecular toxicity. This approach has been used in Quantitative Structure–Activity Relation (QSAR) in drug design and molecule toxicity prediction.^13^ Molecular descriptors can correlate positively or negatively with toxicity. For example, hydrophobicity often shows a positive correlation with toxicity as hydrophobic molecules penetrate cell membranes more easily, while higher PSA reduces membrane permeability and toxicity.^19,20^

However, predicting which combination of the molecular descriptors performs best is still a major challenge. There are different kinds of software that can be used for representing molecules, but it is not the goal of this paper to review all of them. A well-known molecule representation for ML application is the Morgan Fingerprint (MFP) working with structure-similarity identification, and it is available in the python-based package RDkit.^21^ In this work, we have generated the molecular descriptors using both commercial and open source software, AlvaDesc 2.0.14 and MFP, respectively. MFP molecular descriptors were generated with several radius and bit lengths. The standard linear molecule symbols, i.e., SMILES, were used to convert the chemical names to a machine-readable format.

#### AlvaDesc Descriptors

AlvaDesc is commercial software that has generated 4179 descriptors (1D and 2D) for our databases. AlvaDesc provides the 1D descriptors as computed descriptors derived from the chemical formula for example number of atoms, molecular weight, etc., while for the 2D descriptors, they are taken from the representation of the molecule.^22^ The number of descriptors calculated for each AlvaDesc block is shown in Table 1. AlvaDesc is also equipped with built in molecular descriptor reduction, such as constant value and near constant value reduction, pair absolute correlation reduction, etc. By applying these molecular descriptor reductions, the final results for our “All” data set were 1260 descriptors.

**Table 1:** Calculated 1D and 2D Descriptors from AlvaDec’s Software

**no.**	**block of descriptors**	# of descriptors
1	constitutional indices	50
2	ring descriptors	35
3	topological indices	79
4	walk and path counts	46
5	connectivity indices	37
6	information indices	51
7	2D matrix-based descriptors	608
8	2D autocorrelations	213
9	burden eigenvalues	96
10	P_VSA-like descriptors	69
11	ETA indices	40
12	edge adjacency indices	324
13	functional group counts	153
14	atom-centered fragments	115
15	atom-type E-state indices	346
16	pharmacophore descriptors	165
17	2D atom pairs	1596
18	charge descriptors	11
19	molecular properties	26
20	drug-like indices	30
21	MDE descriptors	19
22	chirality descriptors	70
**total**		**4179**

#### Morgan Fingerprint Using the Rdkit Python Package

MFP consists of binary vectors that represent whether a specific fragment or substructure in a chemical structure is present (1) or absent (0). MFP is calculated using Morgan algorithms developed in 1965.^23^ The information is compressed using the algorithm and encoded in a binary vector with a predetermined length defined as bits (512, 1024, or 2048). Bits were used with radius properties to differentiate between fragments. The radius determines how far out from each atom the algorithm will look to define the neighborhood, for example, radius 0 considers only the atom itself, radius 1 includes the atom and all its directly bonded neighbors, radius 2 extends to the neighbors of the neighbors, and so on.^24^ In this study, MFPs with radii 2, 5, and 10 in combination with bits 512, 1024, and 2048 were generated.

### 2.3 Machine Learning

Machine learning is used in this work to facilitate the prediction of toxicity for different organic, inorganic, ionic, and mixture chemicals. Traditionally, toxicity is measured as continuous values and then categorized in different groups ranging from nontoxic to highly toxic. However, changing the prediction problem from predicting continuous values (regression) to predicting discrete values (classification) can result in a loss of information.

**Figure 1:**
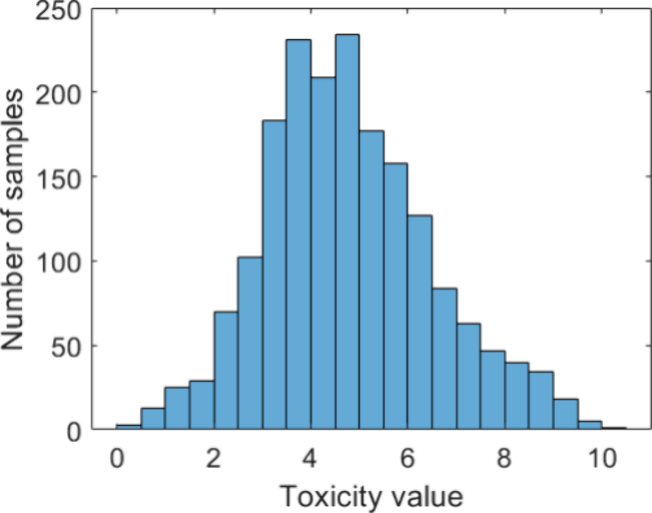
Toxicity value (−Log mol/L) distribution with respect to the number of samples.

Examining the toxicity values used in this study, it is found that they suffer from imbalance distribution; chemicals that are highly toxic or highly nontoxic have a smaller number of examples in the data set compared to chemicals with moderate toxicity (Figure 1). As this work targets chemicals that are safe to be used in EALs, being able to identify highly toxic and nontoxic chemicals is essential. Thus, predicting toxicity in this work is considered and treated as an imbalance regression problem. As imbalance regression is a rather less studied problem in machine learning theory compared to imbalance classification,^25^ this will be the major challenge this work aims to address.

To predict continuous values in a regression problem, we can use either: (a) a single learning algorithm, such as support vector regressor, decision trees, or artificial neural network, among others, or (b) a combination of multiple learners, such as ensemble learners. In ensemble learners, a predefined number of learning models is trained to solve the same prediction problem. However, each learner is trained on a slightly different version of the data and their prediction is combined using voting or averaging approaches.^26^

Generally, ensemble learners can outperform single learners if the set of learners they combine is diverse and have a reasonable performance.^26,27^ Furthermore, ensemble learners can provide several statistical benefits,^28^ such as (a) combining multiple predictors that can compensate for the possible bad prediction a single learner can have for specific target values, (b) provide with a “divide and conquer” strategy where each predictor learn different parts of the data in complex data sets, such as the toxicity data sets used in this work, and (c) can perform better with data fusion, where the data are collected from different sources, which is the case for the data used in this work.

Based on the advantages mentioned above, this paper will explore the application of ensemble learners for imbalance toxicity prediction.

#### 2.3.1 Data

As presented in Section 2.1.2, the final data set consists of 1853 chemicals out of which 1326 are organic compounds. The remaining chemicals are inorganic, ionic, and mixed chemicals. The maximum recorded toxicity value (−Log mol/L) in this data set is 10.1497 (meaning the most toxic substance), while the minimum recorded value is 0.2163 (meaning the least toxic substance); both the mean and standard deviation of the toxicity are 4.7795 and 1.7271, respectively.

Figure 1 shows a histogram with the distribution of the toxicity values (−Log mol/L) in the ECOTOX data set with respect to the number of samples. As can be seen, most of the toxicity values lie in the middle region of the histogram, with 75.72% of the data having a value between 3 and 7 and 90.93% of the data having a value between 2 and 8. The higher and lower toxicity values have a far smaller number of examples. The disproportionate sample distribution among the target values is an issue found in real world regression problems. This problem is referred to as imbalance data distribution,^29^ where certain class(es) in classification problems or certain numeric value interval(s) in regression problems are oversampled in the data. Meanwhile, the remaining classes/target values are undersampled.

Solutions to data imbalancement in machine learning have been often discussed in classification problem scenarios, where multiple solutions were proposed,^30^ such as applying sampling techniques (undersampling and oversampling), using a modified performance measure, and introducing a new weighting scheme that takes into consideration the distribution of samples per class. A noticeable sampling technique that was introduced in 2002 for imbalance data problem is the Synthetic Minority Oversampling TEchnique (SMOTE) sampling,^31^ in which the K-nearest neighbor is used to interpolate new examples from existing minority class data points. This technique was developed for classification problems, and later, it was extended to regression problems using similar sampling techniques.^32,33^ This paper examines this approach to balance the toxicity value distribution.

As mentioned earlier, two software packages were used to generate the molecular descriptors, namely, AlvaDesc and MFP. The number of molecular descriptors generated from AlvaDesc is 1260 descriptors, while for MFP, a grid search was performed to identify the suitable radius and number of bits; these were found to be 10 and 1024 bits, respectively.

This work will compare the impact AlvaDesc and MFP descriptors can have on toxicity prediction since the basis for these two molecular descriptor generators, as well as the nature of these descriptors, is different. MFP focuses on finding a series of binary representations that indicate the absence or presence of a connection within a predefined radius, while AlvaDesc generates continuous descriptors.

#### 2.3.2 Modeling Method

In this paper, a sampling algorithm is used to encounter the imbalance data distribution. This algorithm is Synthetic Minority Over-Sampling Technique for Regression with Gaussian Noise (SMOGN),^33^ which is one of the few sampling methods presented in the literature for imbalance regression problems. It is based on a previous regression sampling technique, known as Synthetic Minority Over-Sampling Technique for Regression (SMOTER) that was presented in ref (32). SMOGN is a distance-based algorithm that facilitates K-Nearest Neighbors (KNN) to add new points in the data through (SMOTER) when the examples are in proximity and add Gaussian noise when the examples are far from each other. Due to this added noise, the distribution of the sampled data is not unique and variations could happen from one run to the other. The resampled data are used to train an ensemble learner.

The ensemble learner used in this study is XGBoost, which is a well-known robust learning algorithm. The performance of the XGBoost will be assessed using three metrics; these are: (1) the coefficient of determination (*R*^2^) that measures the goodness of the regression model fit. It is a linear measure that quantifies the proposition of the variance in the dependent variable (target value/toxicity value) that is predictable from the independent variable (features/descriptors) in a regression model. It ranges between 0 and 1 (the higher its value, the better the model fits the data); (2) the quadratic error measurement using Root Mean Square Error (RMSE), which highlights the model sensitivity to outliers, and (3) the linear error measurement using Mean Absolute Error (MAE).

The methodology followed in this work can be summarized as follows:

1. First, the data are split into 75% training data *D*_tr_ and 25% testing data *D*_ts_.
2. SMOGN is applied to the training data to oversample the minority class and undersample the majority class, resulting in a new data distribution *D*_tr-sampled_.
3. Feature/descriptor selection is applied using mutual information, where only 20% of the features/descriptors (the most informative molecular descriptors) are retained as *D*_tr-reduced_. Using the same setting, feature/descriptor selection is also applied to the testing data resulting in *D*_ts-reduced_.
4. Apply a 10-fold cross validation that is repeated 3 times to estimate the training accuracy of the model. However, as the test accuracy is measured by applying the fully developed model to previously unseen data, we can only measure it once, and we cannot use it to refine the final model’s parameters.
5. XGBoost ensemble with decision trees as base learners is trained and fine-tuned using *D*_tr-reduced_.
6. The obtained model is tested on *D*_tr-reduced_.
7. *R*^2^, RMSE, and MAE are recorded for both training and testing data.

**Figure 2:**
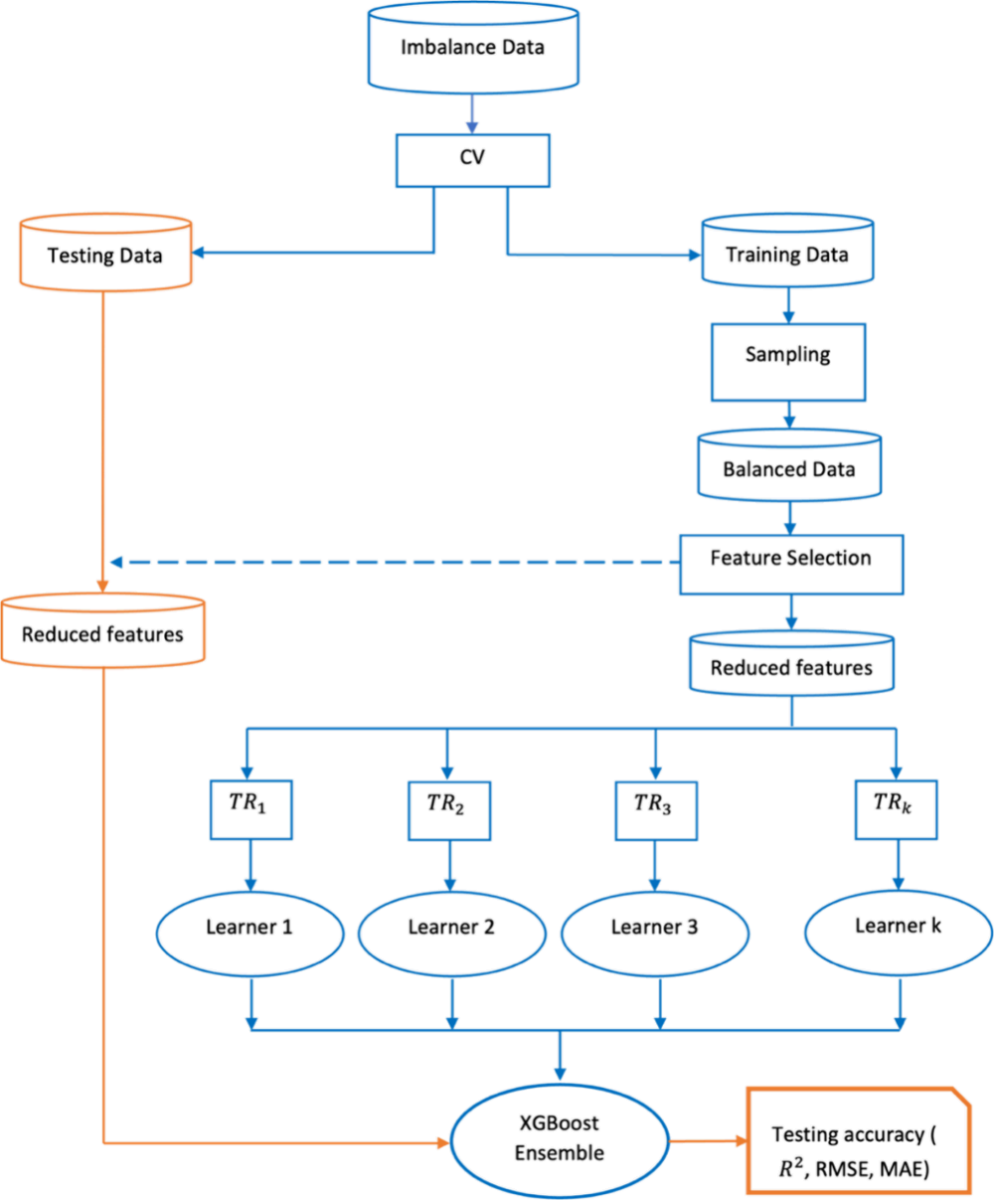
Methodology followed in this work, where CV refers to cross validation and TR refers to the subsamples of the training data. The training phase is highlighted with the blue components of the flowchart, while the testing phase is highlighted with the orange components.

Furthermore, to test the effectiveness of using these sampling techniques, the experiments are repeated using the original data distribution without applying SMOGN. The flowchart presented in Figure 2 illustrates the methodology followed in this work.

Including all features increased the model’s complexity and execution time without significantly enhancing its accuracy. Conversely, utilizing only 20% of the features not only reduced model complexity and the risk of overfitting but also maintained similar accuracy compared to the complete feature set. The results in Table 2 illustrate the performance of our largest data set when using all features versus when only 20% of the features were used.

**Table 2:** Comparing Model Accuracy with and without Applying Feature Selection

data set	feature percentage	train/test	descriptors	***R*^2^**	RMSE	MAE
**All**	20%	train	AlvaDesc	0.9403	0.4172	0.2559
**All**	20%	test	AlvaDesc	0.6589	1.0403	0.7699
**All**	100%	train	AlvaDesc	0.9509	0.3787	0.2379
**All**	100%	test	AlvaDesc	0.6611	1.0324	0.7608

Furthermore, different ratios of 60:40, 75:25, and 80:20 were tested in this study using the largest data set with AlvaDesc descriptors. A comparison of these data split percentages is illustrated in Table 3.

**Table 3:** Comparing the Different Split Ratios for the Training and Testing Data

data set	split ratio	train/test	descriptors	***R*^2^**	RMSE	MAE
**All**	(75:25)	train	AlvaDesc	0.9403	0.4172	0.2559
**All**	(75:25)	test	AlvaDesc	0.6589	1.0403	0.7699
**All**	(60:40)	train	AlvaDesc	0.9606	0.3486	0.2192
**All**	(60:40)	test	AlvaDesc	0.5569	1.1126	0.8090
**All**	(80:20)	train	AlvaDesc	0.9456	0.4048	0.2457
**All**	(80:20)	test	AlvaDesc	0.6158	1.0462	0.7683

Using 60% of the data set for training and 40% for testing resulted in poorer performance compared to the other two split ratios. This is primarily because the learning algorithm had fewer training data from which to learn from. In contrast, the other two cases exhibited comparable performance, with the 75:25 ratio being slightly better. Therefore, the 75:25 ratio was used in this study.

## 3 Results

The methodology discussed above is applied to three data sets: “All” data set, “Clean” data set, and the ITA data set. The aim of using these three data sets is to examine the model performance on (a) different types of chemicals, (b) only organic chemicals, and (c) to compare its performance to previous work on toxicity prediction.

The parameter setting for SMOGN algorithm differs depending on the data set used:

The number of K neighbors considered for sampling using KNN ranged from 3 to 15 depending on the size of the data, where larger data set such as the “All” data set required 15 to sample the data, the “Clear” data set required 7 neighbors, and the ITA data set required only 3 neighbors.
The perturbation added to Gaussian noise (usually takes a value between 0 and 1) ranged between 0.02 and 0.04.
The threshold for performing over/undersampling (usually takes a value between 0 and 1) ranged between 0.2 and 0.4.

Grid search was used to choose the parameter setting of the XGBoost. The final tuned ensemble model had the following characteristics: the XGBoost learners combined were 1000 decision trees, learning rate was 5 × 10^–3^, and to avoid overfitting, the maximum depth of the trees was set to 5, and each tree is trained using 80% of the data and 80% of the descriptors.

### 3.1 Results with SMOGN

Table 4 shows the training and testing accuracies for all three data sets when XGBoost learner is applied with SMOGN sampling. The results are recorded for both AlvaDesc descriptors and MFP descriptors.

**Table 4:** XGBoost Training and Testing Accuracies Measured by *R*^2^, RMSE, and MAE, when SMOGN Is Applied

data set	train/test	descriptors	***R*^2^**	RMSE	MAE
**All**	train	AlvaDesc	0.9403	0.4172	0.2559
**All**	test	AlvaDesc	0.6589	1.0403	0.7699
**All**	train	MFP	0.7890	0.7852	0.5780
**All**	test	MFP	0.4637	1.3014	0.9688
**Clean**	train	AlvaDesc	0.9239	0.4969	0.3670
**Clean**	test	AlvaDesc	0.6764	0.9825	0.7631
**Clean**	train	MFP	0.7245	0.9368	0.7332
**Clean**	test	MFP	0.5863	1.1408	0.9068
**ITA**	train	AlvaDesc	0.8206	0.6903	0.5091
**ITA**	test	AlvaDesc	0.6769	0.9945	0.7709
**ITA**	train	MFP	0.7803	0.7780	0.6050
**ITA**	test	MFP	0.3813	1.3186	0.9993

Generally, XGBoost trained on AlvaDesc descriptors has a better representation of the underlying data than MFP. This is apparent when comparing the coefficient of determination (*R*^2^) for both training and testing for the two descriptors. Furthermore, the training and testing error for AlvaDesc descriptors is lower than MFP in terms of both RMSE and MAE.

However, XGBoost trained on both descriptors suffers from overfitting. Overfitting refers to the case when learning models have high training accuracies, and when tested, their accuracy drops significantly. This can be noticed in the testing results, with the decrease of the *R*^2^ and the increase in RMSE and MAE. Reducing the model complexity by decreasing the number of decision trees that XGBoost combines did not improve the testing accuracy and resulted in a lower training accuracy.

**Figure 3:**
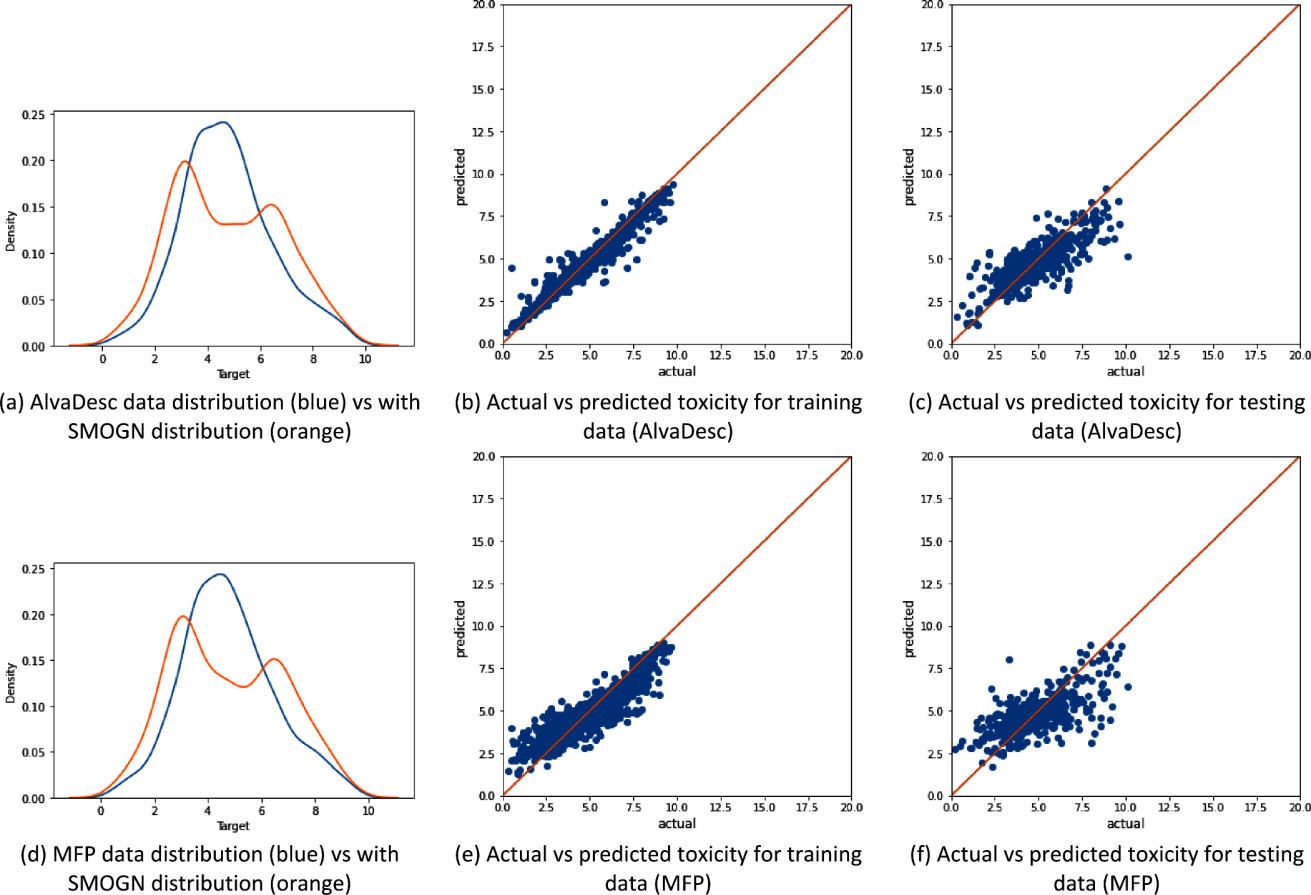
Illustration of the data distribution for the “All” data set (with 1853 samples) and the model performance using SMOGN sampling, where panels (a)–(c) represent AlvaDesc descriptors as the input and panels (d)–(f) represent MFP descriptors as the input.

**Figure 4:**
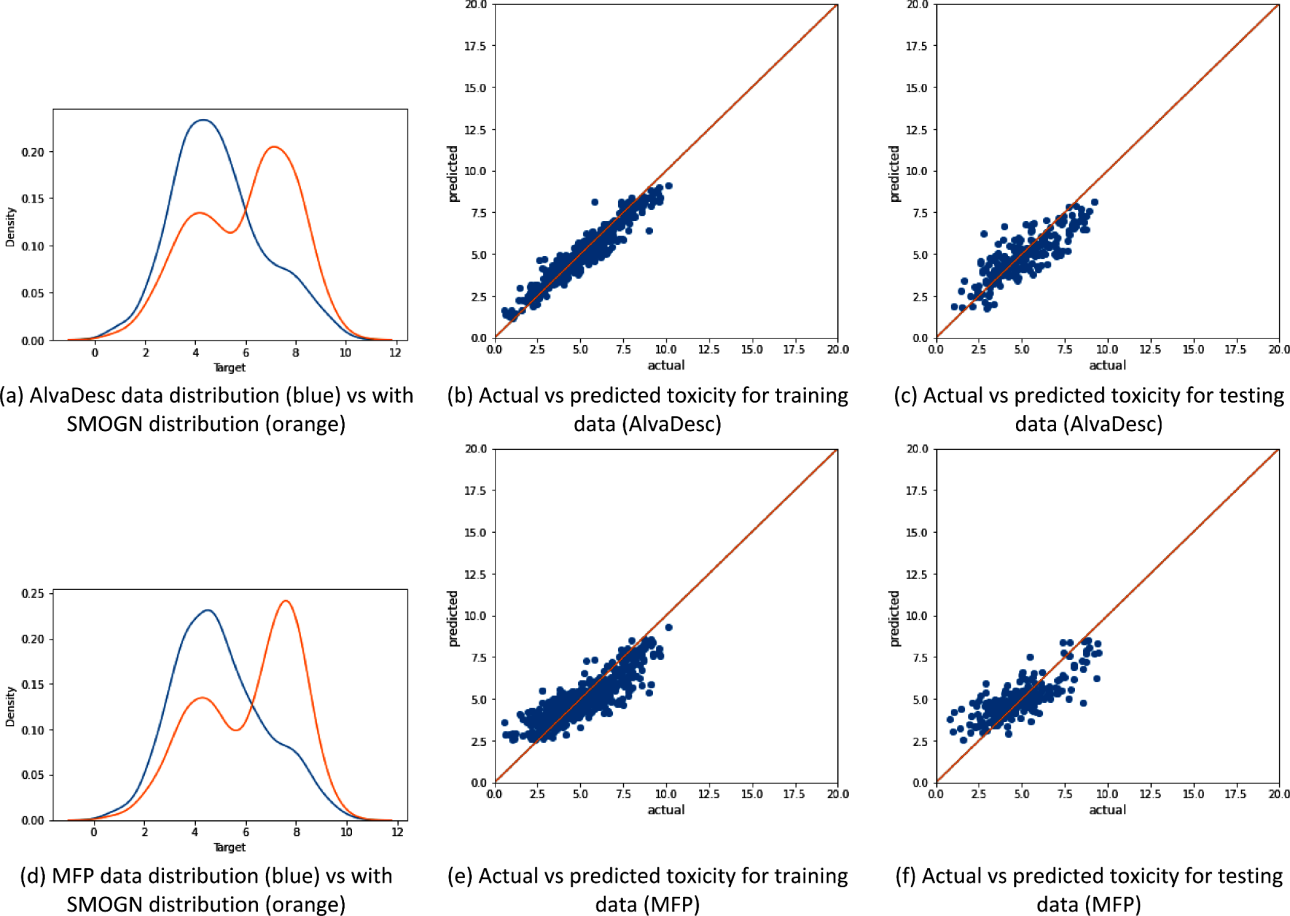
Illustration of the data distribution for the “Clean” data set (with 909 samples) and the model performance using SMOGN sampling, where panels (a)–(c) represent AlvaDesc descriptors as the input and panels (d)–(f) represent MFP descriptors as the input.

**Figure 5:**
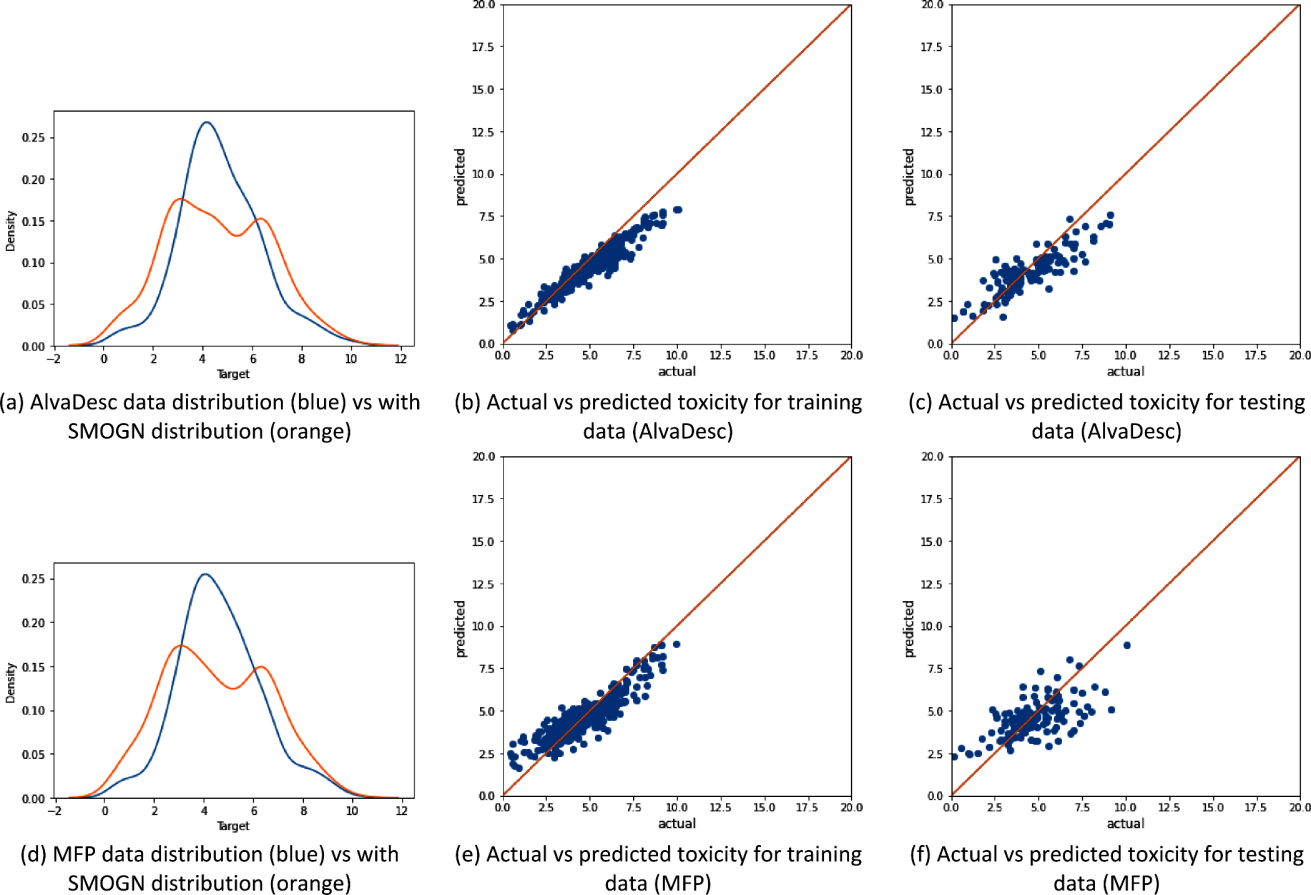
Illustration of the data distribution for the ITA data set (with 546 samples) and the model performance using SMOGN sampling, where panels (a)–(c) represent AlvaDesc descriptors as the input and panels (d)–(f) represent MFP descriptors as the input.

Figures 3–5 show that the data distribution after SMOGN is applied and the relationship between the actual toxicity value and predicted toxicity value for both training and testing for all three data sets.

Figures 3a and 3d show the data distribution when SMOGN is applied to both AlvaDesc and MFP descriptors, respectively. SMOGN performs undersampling for the toxicity values in the range of 3–7 and oversampling on both ends of the toxicity values (<3 and >7). Also, predictions for both training and testing data for AlvaDesc (Figure 3b,c) are better aligned with the actual value compared to that of MFP (shown in Figure 3e,f). This illustrates the higher value AlvaDesc had in terms of *R*^2^ and the lower RMSE and MAE. However, overfitting is apparent for both descriptors, as the XGBoost learner has a wider distribution between the actual and predicted values in testing compared to training.

Figure 4 shows the data distribution and XGBoost performance for the organic molecules. The new distribution found by SMOGN is shown in Figures 4a and 4d, for AlvaDesc and MFP, respectively. In this data set, SMOGN has identified a small increase in the toxicity distribution around value 8 and has incorrectly oversampled this region and undersampled the rest. As it was mentioned in Section 2.3.2, the distribution resulting from applying SMOGN is not unique, and it can be the case where incorrect regions in the data are either over or under sampled. Also, oversampling around the toxicity value 8 using MFP is higher than that with AlvaDesc. Moreover, similar to the previous data set, it can be noticed that AlvaDesc performs better in terms of training and testing predictions and overfitting is present.

Finally, Figure 5 shows the data distribution of the ITA data set and XGBoost performance for both AlvaDesc and MFP descriptors. The data distribution obtained from SMOGN (Figure 5a,e) is more balanced and is better than the one obtained for the “Clean” data set. By examining the training and testing accuracies both in Figure 5 and Table 1, it can be noticed that XGBoost trained on the AlvaDesc descriptor has less overfitting compared to the previous two data sets. However, the overfitting increased when MFP was used.

### 3.2 Results without SMOGN

To test the effect of using sampling to balance the toxicity data, the same experiments are repeated without applying SMOGN. Table 5 shows the training and testing accuracies for both AlvaDesc and MFP for all three data sets.

**Table 5:** XGBoost Training and Testing Accuracies Measured by *R*^2^, RMSE, and MAE, without SMOGN

data set	training/testing	descriptors	***R*^2^**	RMSE	MAE
**All**	train	AlvaDesc	0.9479	0.3976	0.2525
**All**	test	AlvaDesc	0.6499	0.9956	0.7272
**All**	train	MFP	0.8009	0.7633	0.5644
**All**	test	MFP	0.4968	1.2561	0.9709
**Clean**	train	AlvaDesc	0.9851	0.2216	0.1482
**Clean**	test	AlvaDesc	0.6104	1.0571	0.7876
**Clean**	train	MFP	0.8706	0.6347	0.4691
**Clean**	test	MFP	0.5574	1.2231	0.9599
**ITA**	train	AlvaDesc	0.9874	0.1850	0.1319
**ITA**	test	AlvaDesc	0.6268	1.0421	0.7362
**ITA**	train	MFP	0.8851	0.5650	0.4299
**ITA**	test	MFP	0.5431	1.1128	0.8561

Comparing the results in Table 5 with the results when SMOGN was applied (Table 4), no significant differences in the testing and training accuracies were found in the case of using heterogeneous data, such as the “All” data set. However, for homogeneous data sets such as the “Clean” and ITA, SMOGN helped in reducing the gap between testing and training accuracies thus, reducing overfitting. In addition, with AlvaDesc descriptors, there is a small improvement in the test accuracy for these two data sets. Furthermore, XGBoost trained on AlvaDesc descriptors performed better than MFP on all data sets.

**Figure 6:**
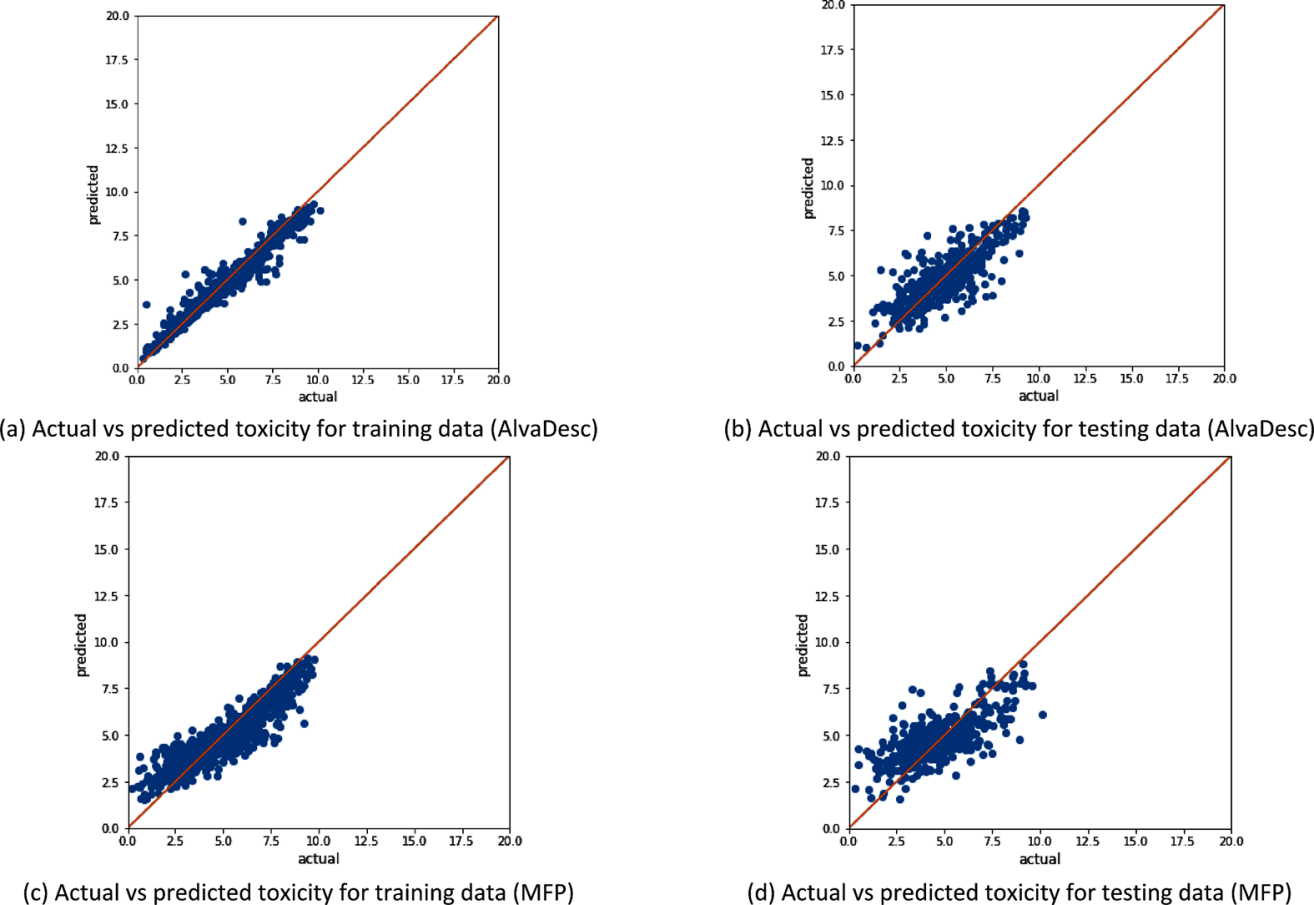
Illustration of the model performance for the “All” data set (with 1853 samples) without SMOGN sampling, where panels (a) and (b) represent AlvaDesc descriptors as the input and panels (c) and (d) represent MFP descriptors as the input.

**Figure 7:**
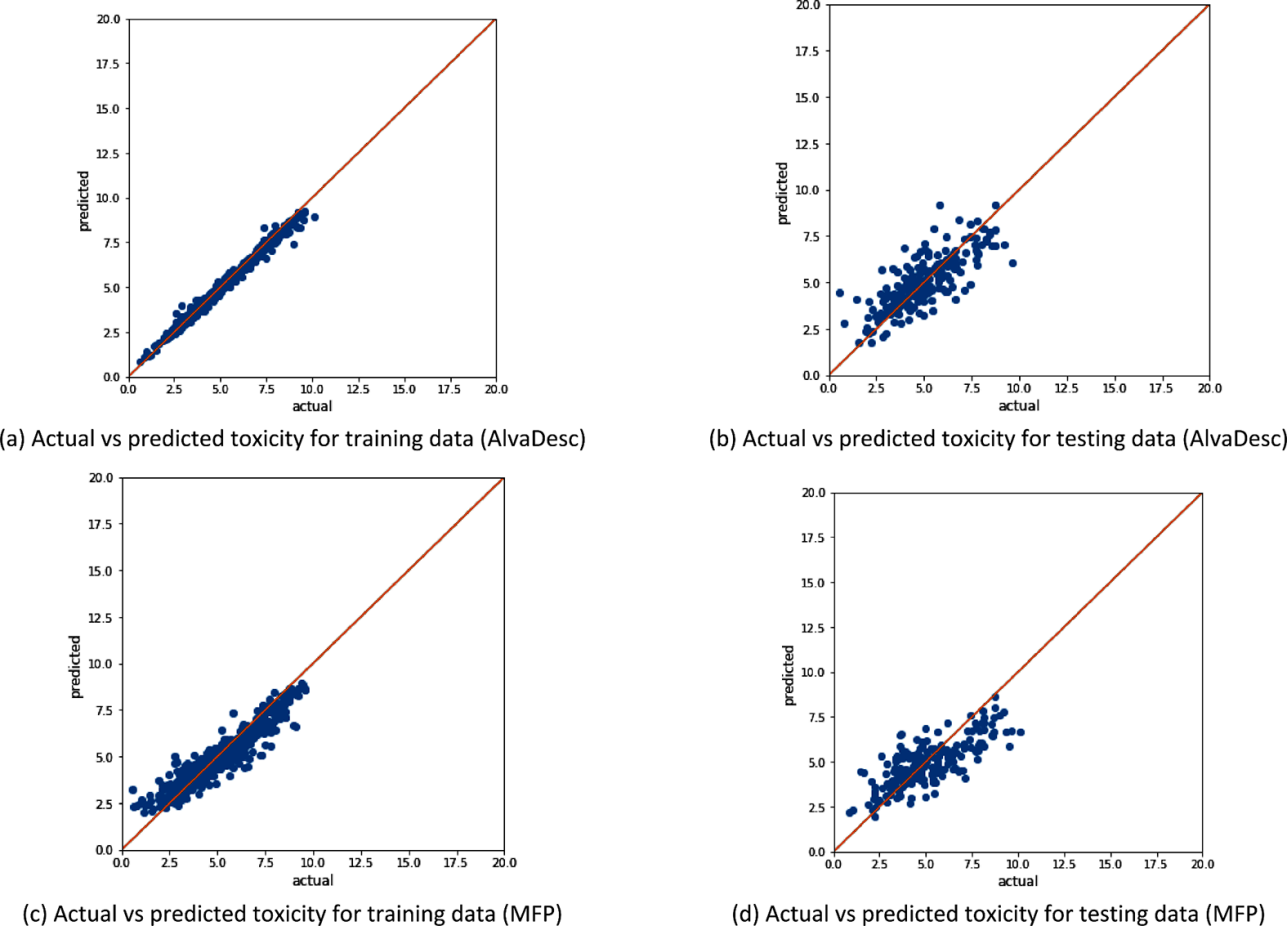
Illustration of the model performance for the “Clean” data set (with 909 samples) without SMOGN sampling, where panels (a) and (b) represent AlvaDesc descriptors as the input and panels (c) and (d) represent MFP descriptors as the input.

**Figure 8:**
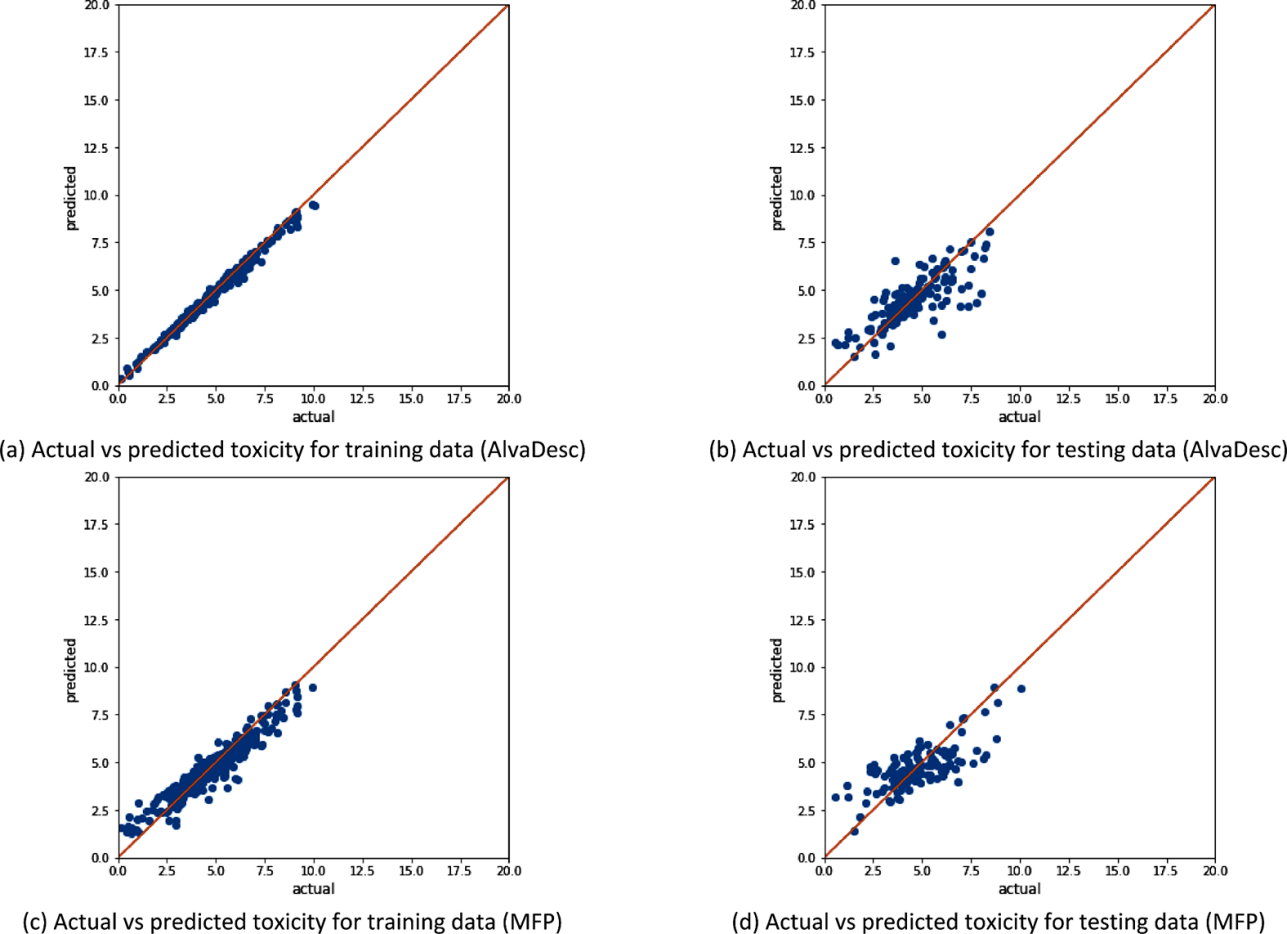
Illustration of the model performance for the ITA data set (with 546 samples) without SMOGN sampling, where panels (a) and (b) represent AlvaDesc descriptors as the input and panels (c) and (d) represent MFP descriptors as the input.

Figures 6–8 examine the relationship between the actual toxicity values and predicted toxicity values for both training and testing for all three data sets. The performance of XGBoost trained on AlvaDesc and MFP descriptors shown in Figure 6 is similar to that obtained in Figure 3. This indicates that applying SMOGN does not improve prediction in heterogeneous toxicity data, which contains organic, inorganic, ionic, and mixed chemicals. For homogeneous data sets like the “Clean” data set, both Figures 7a and 7c show a higher training accuracy than when SMOGN was applied (Figures 4b and 4e). However, the testing accuracies were lower by a small margin. This indicates overfitting when no sampling algorithm is used to balance homogeneous data. Similar to the “Clean” data set, training the XGBoost without using sampling in the ITA data set resulted in a better training accuracy and slightly lower testing accuracy, which indicates overfitting.

## 4 Discussion

### 4.1 Effect of Data Balancing on the Accuracy of the Prediction

The use of the SMOGN sampling technique to balance the toxicity target distribution has impacted the prediction accuracy and how well the model fits the data.

In homogeneous data, such as the “Clean” and ITA data sets, this effect was apparent in terms of the *R*^2^, RMSE, and MAE, especially when AlvaDesc descriptors were used. On the other hand, in heterogeneous data sets such as the” All” data set, SMOGN had a little effect. This could indicate that the use of sampling techniques (such as SMOGN) to balance the target distribution can have a negligible effect in data fusion scenarios, where more than one type of chemical compounds is presented. While for homogeneous data, the use of SMOGN can improve the accuracy and reduce the gap between the training and testing error, thus reducing overfitting.

However, the new target distribution found by SMOGN is not unique. This is due to the Gaussian noise that SMOGN adds when the data points (chemical compounds) are far from each other in the feature/descriptor space. When the data points are in close proximity, SMOGN applies K-nearest neighbors to generate new examples for the undersampled target values. However, when data points are far away from each other, Gaussian noise with a predefined perturbation is added instead. As a result, the newly found target distribution can change each time SMOGN is applied.

In addition, for both MFP and AlvaDesc, balancing the data did not improve the result in the case of the heterogeneous data set, but it slightly helped in reducing the overfitting between training and testing accuracies of the two descriptors.

### 4.2 Effect of the Descriptor Generator (AlvaDesc versus MFP) on Model Accuracy

The effect between molecular descriptor generators (AlvaDesc and MFP) is clearly seen from Figures 3–8 as well as Tables 4 and 5. In all cases, AlvaDesc resulted in better accuracy compared to MFP. AlvaDesc generates a variety of molecular information, such as, constitutional (related to counting atoms and bonds), topological (related to graph invariants of atom bonds), and pharmacophore (related to the statistical significance of the molecular structure–activity correlation). AlvaDesc also provides the calculations of several model-based physicochemical properties such as molecular properties, drug-like and lead-like indices. Twenty-two sets of molecular descriptors generated by AlvaDesc for the data analyzed in this paper are listed in Table 1. This provides AlvaDesc with an advantage with respect to MFP, which relies only on the chosen number of bits in single molecules, discarding any other molecular property. In other words, MFP only requires atomic connectivity information and allows one to compare molecules to evaluate similarity. It is thus to be expected that a predictive model for toxicity may not perform well if only constitutional molecular information is considered by the descriptors in the model.^34^

In addition, the MFP algorithm involves an atom identifier, concatenation and hashing, and fingerprint generation. The concatenated identifiers capture the structural features or substructure of the circular neighborhood around each atom, while hashing generates a unique identifier for the substructure and then maps the concatenated identifier to a fixed-size string of bits. At the end, the fingerprint is represented as a fixed-length binary vector (bit vector), where each bit in the vector corresponds to a specific hashed circular substructure. If the substructure is present in the molecule, the corresponding bit is set to 1; otherwise, it is set to 0. The drawbacks of hashing are collisions and information loss. Collision occurs because the hash function maps many possible substructures to a fixed number of bit positions, meaning that different substructures might map to the same position. In addition, hashing simplifies substructures into fixed-size codes, which may omit some detailed information. These limitations in MFP generation may result in inferior performance compared to AlvaDesc in cases like the one studied in this paper, where many molecules might have similar fingerprint molecular descriptors but very different toxicity.

### 4.3 Comparing Results to Other Toxicity Studies

To evaluate the accuracy, performance, and validity of our model for an imbalance regression problem, we have applied our methodology to a data set published in ref (16). This data set (ITA data set) was thoroughly cured and contains only organic chemical compounds that were used to predict continuous toxicity values. The model introduced in ref (16) uses K-nearest neighbors with a similarity distance metric (based on Mahalanobis distance) to include or exclude different chemicals from the data set, such that, those chemicals with a distance greater than a given threshold are excluded from the prediction. The value of this threshold is optimized using a genetic algorithm. The coefficient of determination *R*^2^ reported for this model was 0.78, which is higher than the *R*^2^ obtained in our proposed ensemble model (0.6769). However, this increase in the *R*^2^ value was due to omitting the examples that are hard to get in the prediction, using the threshold function. Furthermore, the authors reported only the testing accuracy, which does not provide insight into the amount of overfitting observed. Therefore, despite having lower *R*^2^, our model can predict a wider variety of chemicals, being better at generalizing undersampled and sparse regions in the data sets.

### 4.4 Usefulness of This Approach for Lubricant Generation

**Figure 9:**
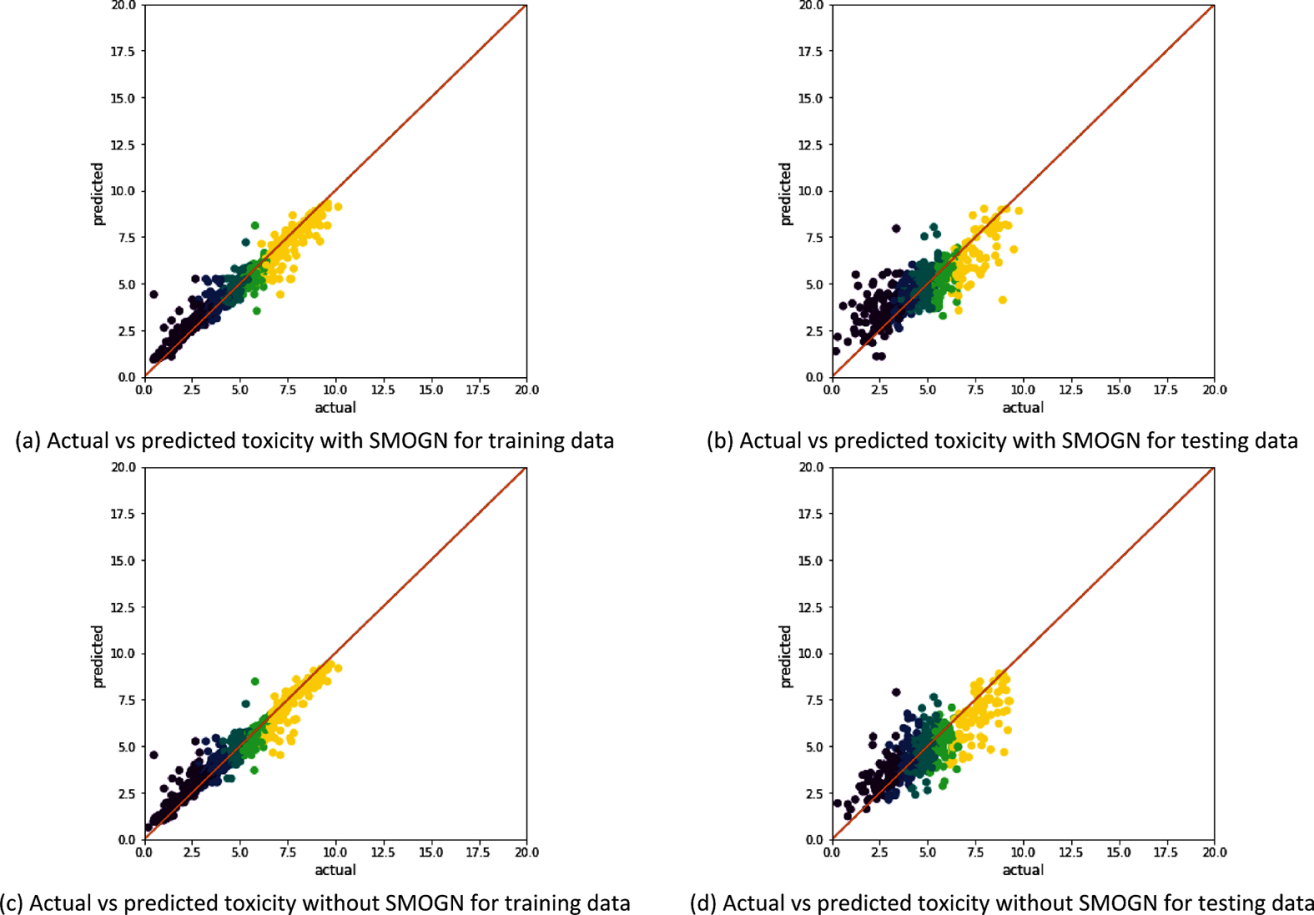
Comparing model performance with AlvaDesc descriptors for the All data set (with 1853 samples) with and without SMOGN sampling, where panels (a) and (b) represent the case when SMOGN used and panels (c) and (d) represent the case without SMOGN. The colors represent the class of the toxicity value (yellow is highly toxic, light green is moderately toxic, dark green is slightly toxic, light blue is practically nontoxic, and dark blue is nontoxic).

This work has shown how challenging it can be to deal with imbalance regression problems. For the set of chemical substances studied in this work, such an effect is especially relevant for undersampled data. In Figure 9, we have plotted the predicted versus the actual values of different toxicity levels for our largest heterogeneous data set (“All” data set) and have compared the models with and without the sampling technique SMOGN. Interestingly, comparing the results in Figure 9 with the number of samples on each toxicity level according to the VGP2013 requirements (Table 6), it is seen that the model has a better prediction on the highly toxic substances (yellow points in Figure 9), where the data contains 624 examples. Meanwhile, it has a slightly worse prediction for nontoxic chemicals (dark blue points in Figure 9), where the data contain only 104 examples.

**Table 6:** Number of Samples in the “All” Dataset Split in the Different Toxicity Levels According to VGP2013^6^

**concentration**^a^	**toxicity level**	# of samples in “All” data set
≥1000 mg/L	nontoxic	104
≥100 mg/L	practically nontoxic	363
10–100 mg/L	slightly toxic	413
1–10 mg/L	moderately toxic	465
≤1 mg/L	highly or very highly toxic	624

a Despite the VGP2013 stating the concentrations of the chemicals in mg/L, in this work, all toxicity values have been calculated and converted to molarity concentrations, which is then transformed into the negative logarithmic scale (−Log mol/L). This is to ensure a positive uniform distribution of the toxicity, as the range of toxicity value measured as mg/L can be wide. Also, this transformation was in line with the curation carried out in ref (16).

The data alignment of different toxicity levels (from highly toxic, yellow, to nontoxic, dark blue) is best for the training data than for the testing data, both with and without SMOGN. Generally, applying SMOGN resulted in a better alignment for toxicity prediction for the substances that lie in between the highly toxic and nontoxic, and larger errors are associated with the two extremes of the toxicity values. This can be a result of the model being trained on artificial chemical substances generated by SMOGN in these regions, leading the model to learn new dependencies between the toxicity value and artificially generated descriptors, which might be imprecise. However, introducing these new generated substances helped the model in encountering noise and outliers, thus reducing overfitting. It should be noted that artificially generated substances using SMOGN improved the model predicting ability of different toxicity levels when homogeneous data were considered.

Nevertheless, the prediction errors obtained are not that big when thinking about lubricant generation. Indeed, for a lubricant to be considered environmentally acceptable, the Vessel General Permit (VGP2013) in Appendix A in ref (6) requires the lubricant to be “minimally toxic”. In the frame of the VGP2013, minimally toxic means a substance that must pass either OECD 201, 202, and 203 for acute toxicity testing, or OECD 210 and 211 for chronic toxicity testing.^6^ More specifically, the LC50 of formulated lubricants must be at least 100 mg/L, and at least 1000 mg/L for greases, two-stroke oils, and all other total loss lubricants. However, when looking at each individual chemical in a lubricant formulation, those chemicals with a concentration below 20 wt % in the formulation can have an LC50 between 10 and 100 mg/L, chemicals with a concentration below 5 wt % in the lubricant can have an LC50 between 1 and 10 mg/L, and chemicals with a concentration below 1 wt % in the lubricant can have an LC50 below 1 mg/L. Interestingly, individual chemicals in additive packages are typically added in the formulation in a concentration well below 20 wt % and only the base oil/lubricant is present in a concentration above 20 wt %. Therefore, having a model that better predicts the most toxic substances is an advantage since this can help in deciding on the maximum concentrations and choices for base lubricants based on the VGP2013 reregulation.

## 5 Conclusions

Handling data imbalance in regression problems is a challenging task. The aim of building a regression model is to perform well in practice and generalize it to previously unseen data. This means that the model should be able to filter out outliers and noise examples in the data. However, when the target value has undersampled regions, these can be viewed as noise by standard regression models and consequently overlooked.

The results of this work indicate that using sampling techniques, such as SMOGN, can improve the performance of the ensemble leaner, like XGBoost, with a small margin for homogeneous data sets (for example only organic molecules). Furthermore, it can reduce overfitting and the model sensitivity to outliers as measured by RMSE. However, sampling techniques can have a limited impact on how well the model predicts different regions within the toxicity distribution. The choice of chemical descriptors can affect how well machine learning models can learn the data, especially when molecular descriptors do not provide enough information about molecules. In this study, XGBoost trained on AlvaDesc descriptors had a higher accuracy compared to when it was trained on MFP because MFP is typically limited to atomic connectivity and similarities.

In the literature, the problem of imbalance regression has been investigated in terms of using sampling techniques, such as the SMOGN sampling technique used in this study, or by considering a modified evaluation metric, or by introducing a weighting scheme that assigns higher weights for samples in undersampled regions. An alternative promising approach is to train local models on data regions to generate local experts. This can help in modeling and assigning higher importance to undersampled regions in the data without altering the original data distribution and/or adding artificially generated samples. Previous studies have suggested that locality in learning can improve both the accuracy and robustness of ensemble models.^35,36^

Despite the prediction errors found in this work, the results are useful for lubricant generation since according to the VGP2013, slightly, moderately, highly, or even very highly toxic chemical substances can still be used in lubricant formulations when they are present in less than 20 wt %. Those levels of toxicity are the ones that are more accurately predicted in this work, and it can therefore help lubricant formulators to use the right concentration for new substances according to their predicted level of toxicity without the need for performing actual testing.
